# Suitability of Direct Resin Composites in Restoring Endodontically Treated Teeth (ETT)

**DOI:** 10.3390/ma17153707

**Published:** 2024-07-26

**Authors:** Markus Heyder, Stefan Kranz, Bruno Wehle, Ulrike Schulze-Späte, Julius Beck, Christoph-Ludwig Hennig, Bernd W. Sigusch, Markus Reise

**Affiliations:** 1 Department of Conservative Dentistry and Periodontology, Center of Dental Medicine, Jena University Hospitals, 07743 Jena, Germanymarkus.reise@med.uni-jena.de (M.R.); 2 Department of Orthodontics, Center of Dental Medicine, Jena University Hospital, 07743 Jena, Germany

**Keywords:** dental adhesives, elastic modulus, endodontic treatment failure, post-endodontic therapy, root canal, tooth fracture, shear bond strength, three-point bending flexural test

## Abstract

(1) Background: The in vitro study aimed to investigate mechanical characteristics of resin composites and their suitability in direct restauration of endodontically treated teeth (ETT). (2) Methods: 38 endodontically treated premolars with occlusal access cavities were directly restored using the following resin composites and adhesives: Tetric Evo Ceram^®^ + Syntac classic^®^ (*n* = 10), Venus Diamond^®^ + iBond Total-Etch^®^ (*n* = 10), Grandio^®^ + Solobond M^®^ (*n* = 9), Estelite^®^ Sigma Quick + Bond Force^®^ (*n* = 9). After thermocycling, the elastic modulus, shear-bond-strength, fracture load (Fmax) and fracture mode distribution were evaluated. Statistical analysis: one-way ANOVA, *t*-test, Kruskal–Wallis test; *p* < 0.05. (3) Results: Grandio^®^ showed the highest E-modulus (15,857.9 MPa) which was significant to Venus Diamond^®^ (13,058.83 MPa), Tetric Evo Ceram^®^ (8636.0 MPa) and Estelite^®^ Sigma Quick (7004.58 MPa). The highest shear-bond-strength was observed for Solobond M^®^ (17.28 MPa), followed by iBond^®^ (16.61 MPa), Syntac classic^®^ (16.41 MPa) and Bond Force^®^ (8.37 MPa, *p* < 0.05). The highest fracture load (Fmax) was estimated for ETT restored with Venus Diamond^®^ (1106.83 N), followed by Estelite^®^ Sigma Quick (1030.1 N), Tetric Evo Ceram^®^ (1029 N) and Grandio^®^ (921 N). Fracture-mode distribution did not show any significant differences. (4) Conclusions: The observed resin composites and adhesives show reliable mechanical characteristics and seem to be suitable for direct restoration of endodontically treated teeth.

## 1. Introduction

Due to a loss in structural integrity, endodontically treated teeth (ETT) show reduced biomechanical stability and are therefore prone to greater failure risks. In particular, expanded access cavities cause increased tooth flexibility, resulting in cusps deflection and fracture propagation [1,2,3,4]. In order to prevent fracture-based events, restorative concepts mainly aim to support and conserve the remaining tooth tissue. Those concepts include build-ups with post-and-core designs, reconstructions with partial or total crowns as well as restorations with resin composites, amalgam or ceramics [5,6,7,8].

Due to the great availability of adhesive techniques, restorative concepts on ETT have changed significantly. In daily practice, ETT are increasingly being restored using direct resin-composites, often in combination with fiberglass posts [1,5,9,10]. Although evidence is still lacking, the clinical use of resin composites is safe and shows great potential [11,12,13,14,15]. In accordance with the already published data, it is generally assumed that preparing sound tooth tissue in order to place a full contour metal crown may also not be suitable anymore in a contemporary dental practice aided by adhesive dentistry.

The use of resin composites enable clinicians to adhesively restore teeth that would otherwise require extensive preparation to increase mechanical retention [9]. Clinically, resin composites are applied by either a direct or indirect approach. While direct composite restorations are fabricated in a single appointment, indirect reconstructions are produced outside the oral cavity with greater control over the anatomy. It was shown that in the short-term (2.5 to 3 years), evidence suggests that there is no difference in tooth survival or restoration quality between direct and indirect resin composite restorations [10]. Overall, survival rates between 80 and 90% were observed for ETT restored with direct resin-composites fillings after 3 years of wear [16]. In this regard, restored endodontically treated maxillary premolars with various materials (conventional composite resin with or without horizontal fiber post, bulk-fill composite, ceramic inlay) showed fracture resistance similar to those of sound teeth [17]. In this context, it is important to note that the choice of the restorative technique also depends on the remaining dental tissue. Composite direct restorations are suitable for simple occlusal endodontic access cavities. However, restorative techniques may change in accordance with the remaining cusp wall thickness, margin preparation design and type of material selected [18,19,20].

Today, there are many different resin composites and adhesives available. However, information regarding their suitability for restoring endodontically treated posterior teeth is still lacking. Therefore, the present in vitro study aimed to investigate the mechanical characteristics of ETT that were adhesively restored by direct occlusal resin composite fillings. The null hypothesis (H0) assumes that there are no significant differences in biomechanical properties and fracture strength between the tested materials.

## 2. Materials and Methods

### 2.1. Resin Composites and Adhesives

In the present investigation the resin composites Venus Diamond^®^ (Heraeus Kulzer^®^, Hanau, Germany), Grandio^®^ (VOCO^®^, Cuxhafen, Germany), Tetric Evo Ceram^®^ (Ivoclar Vivadent^®^, Schaan, Liechtenstein), and Estelite^®^ Sigma Quick (Tokuyama Dental^®^, Tokyo, Japan) were used. Material specifications are listed in Table 1.

As recommended by the manufacturer, each resin composite was used in combination with an appropriate adhesive. The following adhesives were applied: Syntac classic^®^ (Ivoclar Vivadent^®^, Schaan, Liechtenstein), iBond Total-Etch^®^ (Heraeus Kulzer^®^, Hanau, Germany), Solobond M^®^ (VOCO^®^, Cuxhafen, Germany), and Bond Force^®^ (Tokuyama Dental^®^, Tokyo, Japan). The applied adhesives are summarized in Table 2. The adhesives Syntac classic^®^, iBond total etch^®^ and Solobond M^®^ required an additional “etch&rinse” step, which involved the application of 35% ortho-phosphoric acid (Vococid^®^, VOCO^®^, Cuxhafen, Germany). Resin composite and adhesive combinations as well as the required conditioning measures are summarized in Table 2.

### 2.2. Three-Point Bending Flexural Test (Elastic Modulus) of Resin Composites

From each resin composite, 12 test specimens (25 × 2 × 2 mm) were fabricated in accordance with DIN EN ISO 4049:2000 (DIN 2019) [21] using a brass metal mold. After application of the respective resin composite, the mold was covered by a transparent coverslip and the composite samples were photo-polymerized for 180 s on each side using the Dentacolor XS device (Kulzer, Wehrheim, Germany).

After storage in water for 24 h, the specimens were transferred to a table-top testing machine (Z005, Zwick/Röll, Ulm, Germany) and subjected to fracture load at a speed of 1 mm/min. The span length was set to 20 mm.

### 2.3. Compression–Shear Test (Shear Bond Strength) of Resin Composite Samples Bonded to Dentin Discs

To examine the shear bond strength, extracted third molars were used. The study was approved by the local ethic committee (Ethic Committee, Medical Faculty, Friedrich-Schiller University, Bachstraße 18, 07743 Jena, Germany; ID: 2019-1401_1-Material). Written informed consent was given. The collected teeth were stored in 2% CHX-solution until use. For testing, teeth were cleaned, embedded in clear self-polymerizing resin (Paladur^®^, Heraeus Kulzer^®^, Hanau, Germany) and ground horizontally until dentin was exposed. The dentin surfaces were then conditioned using dentin adhesives and 35% ortho-phosphoric acid according to the manufacturer instructions (Table 2). Light-curing (700 mW/cm^2^, 40 s) was carried out using a calibrated halogen-driven light-curing unit (Elipar Highlight, 3M ESPE^®^, Seefeld, Germany).

A cylindrical metal brass mold (4.0 mm ∅ × 2 mm) was then placed on top of the conditioned dentin surface and filled with the respective resin composite (Table 2). Subsequently, all specimens were covered by a transparent plastic foil and light cured for 60 s each. The mold was removed, and each specimen was light cured for another 60 s. For each resin composite and adhesive combination (Table 2), 18 samples were fabricated.

After storage in water for 24 h, the test specimens were subjected to loading using a table-top testing machine (Z005, Zwick/Röll, Ulm, Germany) at a speed of 1 mm/min parallel to the dentin surface until the composite specimen sheared off. The maximum shear off force (Fmax) was recorded.

### 2.4. Endodontic Treatment and Direct Occlusal Restoration

A total of 38 single-rooted premolars were assigned to 4 different composite/adhesive groups:

Group 1: Tetric Evo Ceram^®^ and Syntac classic^®^    (*n* = 10)

Group 2: Venus Diamond^®^ and iBond Total-Etch^®^  (*n* = 10)

Group 3: Grandio^®^ and Solobond M^®^     (*n* = 9)

Group 4: Estelite^®^ Sigma Quick and Bond Force^®^   (*n* = 9)

Premolars were extracted because of orthodontic reasons. All patients were informed, and written consent was given. The study was approved by a local ethic committee (Ethic Committee, Medical Faculty, Friedrich-Schiller University, Bachstraße 18, 07743 Jena, Germany; ID: 2019-1401_1-Material).

At first, crowns were trepanated in the center proportion of the occlusal face using a high-speed contra-angle handpiece with a ratio of 1:5 and medium-grain (100 µm) cylindrical burs (Komet^®^ Dental, Lemgo, Germany). The diameter of the cylindrical occlusal access cavity was 5 mm which was checked with a caliper (Munich model/Dentaurum^®^, Isprunge, Germany). After removal of the remaining pulp tissue, root canals were mechanically enlarged to full working length using hand-held files up to size ISO 55 (ISO 3630-1:2019 [22]). Each enlargement step was accompanied by an intracanal rinse with 10 mL of 0.5% NaOCl. After a final rinse with 17% EDTA, all canals were dried using paper points and filled in lateral condensation technique with gutta-percha and sealer (AH Plus, Densply DeTrey, Konstanz, Germany). All ETT were subjected to radiographic examination in order to verify the result of the root canal obturation procedure.

Next, the cavity floor was lined with glass ionomer cement (Ketac™ Bond, 3M ESPE^®^, Seefeld, Germany) to ensure a universal composite thickness of 5 mm in height.

All access cavities were cleaned and conditioned as recommended by the specific manufacturer (Table 2). Subsequently, cavities were filled with resin composites in increments of 2 mm. Each increment was light cured for 40 s using the light-curing unit Mini LED OEM (Satelec/Acteon, Mettmann, Germany) at 1100 mW/cm^2^. Finally, the surface of each direct restoration was finished and polished using rotary instruments (Komet GmbH, Besigheim, Germany).

### 2.5. Occlusal Fracture Load (Fmax) and Fracture Patterns of Endodontically Treated Teeth Restored with Direct Resin Composite Fillings

**Figure 1:**
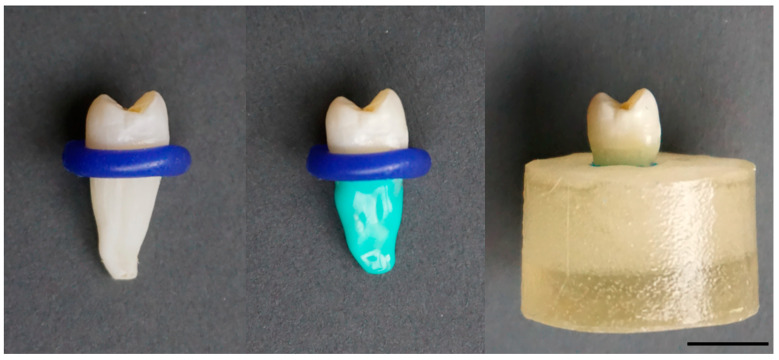
ETT premolar restored with occlusal direct resin composite filling. The enamel–cementum junction was covered by a ring of wax (**left**). Root surface was coated with A-silicon for simulating the biomechanical behavior of the periodontium (**middle**). ETT was embedded into transparent resin (**right**). Scale bar 1 cm.

At first, restored ETT were subjected to thermocycling (25,000 cycles, 5–50 °C). Subsequently, the root surfaces were covered with A-silicon (Flexitime^®^, Heraeus Kulzer^®^, Hanau, Germany) and embedded into transparent resin (Paladur^®^, Heraeus Kulzer^®^, Hanau, Germany) with a distance of 1 mm to the enamel–cementum junction (Figure 1). The silicon coating was used for simulating the biomechanical behavior of the periodontium.

**Figure 2:**
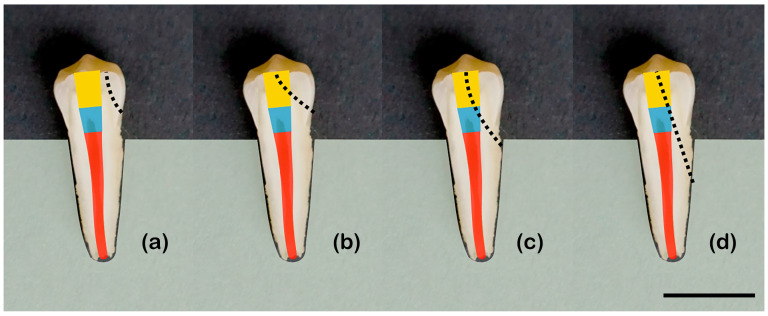
Fracture patterns. Endodontically treated premolars with root canal filling (red), glasionomer cement lining (blue) and direct resin composite restoration (gray). Dotted lines indicate fracture course: (**a**) fractures of the coronal tooth structure (grade 1); (**b**) fractures involving the coronal tooth structure and cohesive filling failure (grade 2); (**c**) fractures involving the tooth structure, cohesive and/or adhesive filling failure with root involvement (grade 3); and (**d**) severe crown and root fracture (catastrophic fracture, grade 4). Scale bar 1 cm.

The center of the occlusal face was subjected to axial loading until fracture using the table-top testing machine (Z005, Zwick/Röll, Ulm, Germany) at a speed of 1 mm/min. Fmax and the fracture pattern was documented. For analyzing the fracture course, a 4-graded score was applied (Figure 2) that was introduced by Burke et al. [23] and modified by Soares et al. [24]:

Group 1. fractures involving a small portion of the coronal tooth tissue.
Group 2. fractures involving a small portion of the coronal tooth tissue and cohesive failure of the restoration.
Group 3. fractures involving the tooth tissue, cohesive and/or adhesive failure of the restoration, with root involvement that can be restored in association with periodontal surgery.
Group 4. severe root and crown fracture, which determine extraction of the tooth (catastrophic fracture).

### 2.6. Statistical Analysis

Results of the three-point bending and shear tests were analyzed using one-way ANOVA and Students’ *t*-test. Significant differences among the fracture categories were evaluated by the Kruskal–Wallis test. For analyzation, SPSS version 13 was used. Significance was obtained for *p* < 0.05.

## 3. Results

In general, as shown by the results, all observed materials seem to be suitable for stabilizing endodontically treated teeth (ETT).

### 3.1. Three-Point Bending Flexural Test (Elastic Modulus) of Resin-Composites

**Figure 3:**
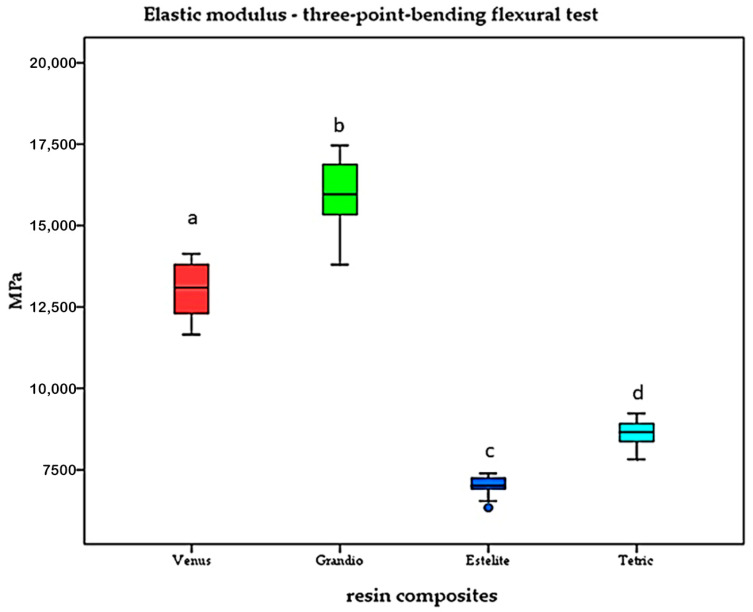
Elastic modulus of resin composites. Significance is marked by different letters (*p* < 0.05).

The highest elastic modulus was recorded for Grandio^®^ (15,857.9 ± 1269.9 MPa), followed by Venus Diamond^®^ (13,058.83 ± 845.36 MPa) and Tetric Evo Ceram^®^ (8636.0 ± 420.4 MPa). Estelite^®^ Sigma Quick showed the lowest elastic modulus (7004.58 ± 313.82). Results of the three-point bending flexural test are presented in Figure 3. Between all groups, significance was determined (*p* < 0.05).

### 3.2. Compression–Shear Test (Shear Bond Strength) of Resin Composite Samples Bonded to Dentin Discs

**Figure 4:**
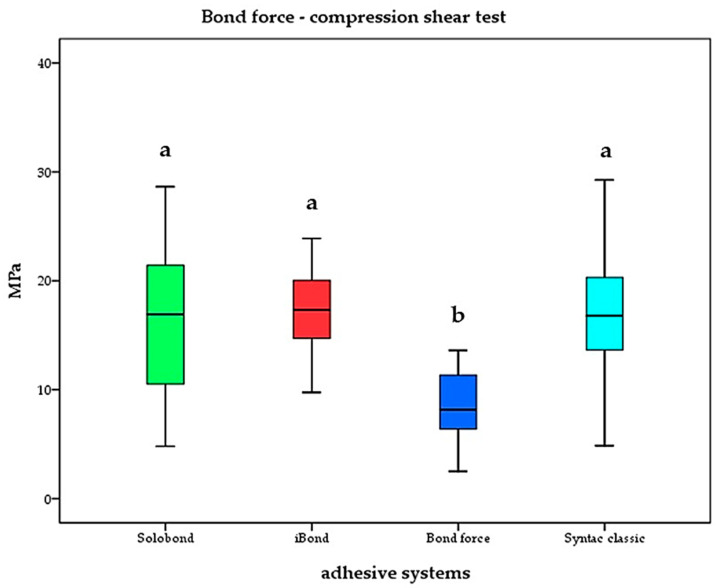
Shear bond strength of the applied adhesives. Significance is marked by different letters (*p* < 0.05).

The highest shear bond strength was recorded for Grandio^®^ in combination with Solobond M^®^ (17.3 ± 4.1 MPa), followed by Tetric Evoceram^®^ with Syntac classic^®^ (16.6 ± 6.2 MPa, *p* = 0.705) and Venus diamond with iBond^®^ (16.4 ± 6.8 MPa, *p* = 0.646). The lowest shear bond strength was analyzed for Estelite^®^ Sigma Quick in combination with Bond Force^®^ (8.4 ± 3.1 MPa). Results are shown in Figure 4. In correlation to Table 2, only the adhesive system is named in Figure 4. Intergroup comparison showed significance only for Estelite^®^ Sigma Quick in combination with Bond Force^®^ (*p* < 0.05).

### 3.3. Occlusal Fracture Load (Fmax) and Fracture Patterns of Endodontically Treated Teeth Restored with Direct Resin Composite Fillings

**Figure 5:**
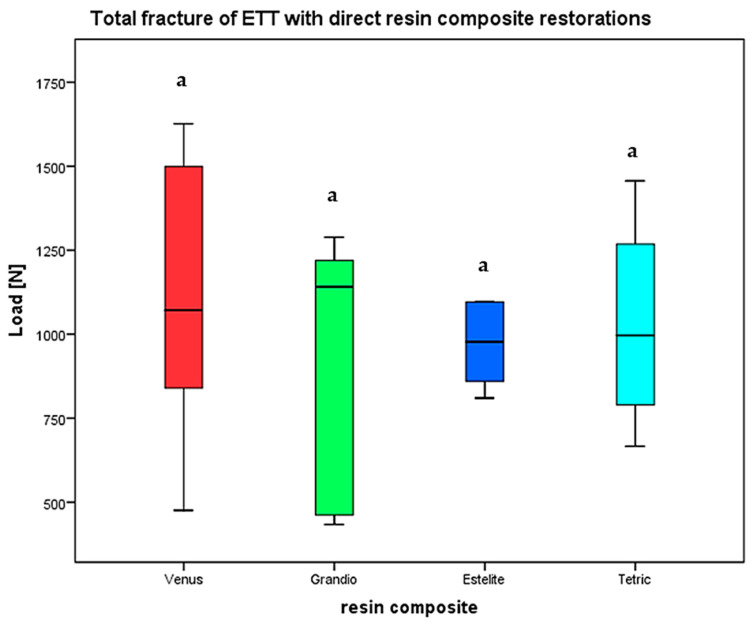
Fracture load (Fmax) of ETT with direct resin composite restorations. Significance is marked by different letters (*p* < 0.05).

Fmax was recorded that caused total fracture of the restored ETT after occlusal loading. Distribution of the respective fracture mode/grade was also observed. The highest fracture load was found for ETT with access cavities restored with Venus Diamond (1106.83 ± 434.4 N), followed by direct restoration with Estelite Sigma Quick (1030.1 ± 242.1 N) and Tetric Evoceram (1029 ± 289 N). ETT restored with Grandio showed the lowest fracture resistance (921 ± 391.9). There was no significant difference between all groups (*p* = 0.851) determined by the Kruskal–Wallis test (Figure 5).

**Figure 6:**
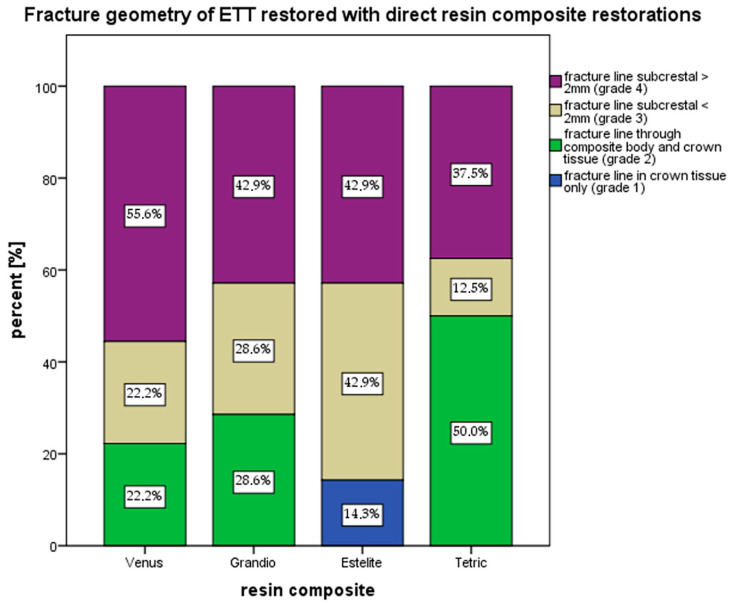
Fracture mode distribution.

For ETT directly restored with Venus, Grandio and Estelite, the majority of fractures were of grade 4 which was the most unfavorable course (Figure 6). Grade 3 fractures were also commonly observed (Estelite 42.9%, Grandio 28.6%, Venus 22.2%), with the lowest proportion for Tetric Evoceram (12.5%).

Grade 2 was observed for Tetric Evoceram (50%), Grandio (28.6%) and Venus Diamond (22.2%). Estelite was the onliest material that showed grade 1 fractures.

Between the single fracture categories, no significant differences were determined (*p* = 0.786).

## 4. Discussion

The aim of the present investigation was to evaluate whether various resin composites in combination with their recommended adhesives differ in their suitability for direct restoration of ETT.

As part of the study design, the mechanical properties (elastic modulus, shear bond strength) were examined first. As the results show, the elastic moduli obtained were close to the values already specified by the respective manufacturer. Only for Estelite^®^ Sigma Quick, a lower value was recorded (7004.58 vs. 8600 MPa) in the present investigation. In contrast to these results, a different research group reported an even higher elastic modulus (9600 MPa) for Estelite^®^ Sigma Quick [25].

However, in order to ensure sufficient biomedical function, the elastic modulus should be close to that of dentin, which is in the range of 15,000–25,000 MPa [26,27,28]. From all tested resin composites, only Grandio^®^ (15,857 MPa) was close to this value. The elastic modulus observed for Grandio^®^ in the present investigation was also confirmed by other authors [29,30].

In the present study, values for Venus Diamond^®^ (13,058.83 MPa; manufacturer’s specification: 12,600 MPa) and Tetric Evoceram^®^ (8636 MPa; manufacturer specification: 10,000 MPa) can be grouped between those of Grandio^®^ and Estelite^®^.

Compared to these results, other research groups reported elastic moduli of 9220.6 MPa for Venus Diamond^®^ and 6034.4 MPa for Tetric Evoceram^®^ [31].

In the present investigation, fabrication and testing of the specimens followed DIN EN ISO 4049:2000. In this regard, the Dentacolor XS device (Kulzer, Wehrheim, Germany) was used for photopolymerization which ensured a sufficient degree of conversation. This means that any loss in mechanical stability due to an inadequate polymerization process can be ruled out.

In the present investigation, all selected resin composites were bonded to dentin samples by their recommended adhesives. As shown by the results of the compression–shear test, no significant differences were detected between iBond^®^ (16.4 MPa), Syntac classic^®^ (16.6 MPa) and Solobond M^®^ (17.3 MPa).

There were also no significant differences between the multi-step adhesive (Syntac classic^®^) and the one-bottle applications (iBond^®^, Solobond M^®^). All tested etch-and-rinse adhesives are in the range of values already known [32,33,34].

However, the only all-in-one adhesive Bond Force^®^ showed the lowest values (8.4 MPa) which was significant to all other values that were examined. The low bond strength that was obtained for Bond Force^®^ in the present study was also confirmed by other authors [35]. Additional selective acid conditioning might have caused an increase in bond strength.

In comparison to the etch-and-rinse adhesives, Bond Force^®^ is of inferior mechanical characteristics in general [36,37,38]. Today, etch-and-rinse adhesives are still superior to self-etch adhesives in terms of their shear bond strength [39,40,41].

As part of the present investigation, the ability of resin composites to stabilize ETT was observed. In detail, resistance to occlusal loads and information regarding the fracture patterns were obtained.

In regard to the maximum fracture load (Fmax), only minor differences between the single materials were found, which were statistically not significant. The lowest fracture resistance was analyzed for ETT restored with Grandio^®^ (920.92 N), while the highest fracture strength was determined for Venus Diamond^®^ (1106.83 N).

In comparison, a different study group examined resistance to axial loads up to 999.6 N when endodontically treated premolars were restored directly with the resin composite Filtek Z350 XT. The same authors reported resistance of sound premolars (controls) up to 949.6 N, while for direct resin composite restorations in combination with horizontal glass fiber posts maximum loads of up to 934.5 N were obtained [17]. In the present investigation, sound premolars were not analyzed as control. Therefore, the mentioned value is to be used for reference. This might also be seen as a limitation and should be taken into account in future observations.

Additionally, it was found that indirect restorations with ceramics (inlays) caused resistance to occlusal loads up to 856.7 N only [17]. In another study using the resin composites Filtek Z250 and Tetric Ceram, fracture strengths of 1269.1 and 1130 N were observed for direct restoration of endodontically treated premolars. The same authors also discovered a fracture load of 1276.1 N for sound premolars [42].

In the present investigation, the highest values were received for restorations with Venus Diamond (1106.83 ± 434.4 N), followed by Estelite (1030.1 ± 242.1 N) and Tetric Evoceram (1029 ± 289 N).

In a recent in vitro study, CAD/CAM manufactured endocrowns, onlays and inlays fabricated from the resin-based nanoceramic Cerasmart^®^ (GC cooperation, Tokyo, Japan) revealed fracture values of 1300.53, 930.70 and 766.90 N in endodontically treated molars with MOD cavities [43]. In endodontically treated premolars the maximum fracture force ranged in between 1119 and 968 N for post-based reconstructions and 859 N for direct restorations using the microhybrid resin composite Charisma^®^ (Heraeus Kulzer, Hanau, Germany) [6]. In comparison, a maximum fracture load of 1106.83 ± 434.4 N was estimated in the present study for occlusal restorations with Venus Diamond^®^.

In the present study the respective fracture patterns were also analyzed. Therefore, a four-graded classification scale was applied [23,24]. As shown by the results, most catastrophic fractures (grade 4) occurred commonly in premolars that were restored with Venus Diamond^®^ (55.6%) and Grandio^®^ (42.9%). Grade 1 fractures (minor fractures in the crown tissue only) were observed for Estelite Sigma Quick (14.3%) only, while restorations with Tetric Evo Ceram were mainly afflicted by grade 2 fractures (50%).

In a similar study, ETT were restored with the resin composite Filtek Z250 in combination with different base materials. Fractures occurring at or above the cementum-enamel junction (CEJ) were grouped together to represent ‘favorable’ fractures (51.3%), and fractures below the CEJ were classified as ‘unfavorable’ (48.7%). It was found that there was no significant association between the proportions of favorable or unfavorable fractures [44]. In the present study, no significant differences in the fracture distribution patterns were found.

It was demonstrated that restorations with fiber-reinforced resin composites showed an increase in fracture resistance and have been supposed for restoring endodontically treated teeth in high-stress-bearing zones. The random fiber orientation in those types of composites effectively prevent polymerization shrinkage, crack propagation, and lead to an even distribution of stress. Samples restored with fiber reinforced composites also exhibited favorable fracturs, that can sufficiently be repaired [45].

In a recent study, direct cavity restoration using the resin composite universal G-aenial A‘CHORD (GC cooperation, Tokyo, Japan) caused the most catastrophic fractures, whereas additional application of the so-called wallpapering technique led to high fracture resistance and increased proportion of repairable fractures [46].

In the present study, a GIC lining was applied on the root canal orifice. In accordance with the crown anatomy, the thickness of the base was different among the test specimens. GIC was used to ensure a standardized resin composite thickness of 5 mm. The base lining might also have an influence on the fracture resistance as well as fracture geometry. This fact is controversially discussed in the literature and still needs to be examined in detail [44,47,48,49]. In this regard it was also shown that a GIC base is beneficial in reducing strain and marginal leakage and therefore it is recommended when endodontically treated teeth undergo direct restoration with the resin composite [50].

In summary, results of the present investigation have shown that endodontically treated premolars with occlusal access cavities can sufficiently be restored and stabilized with direct resin composite fillings. Besides significant differences in the elastic modulus and shear bond strength, no significance was observed for the maximum fracture load and fracture course distribution. In this regard, H0 needs to be rejected.

## 5. Conclusions

In conclusion, the observed modern resin composites in combination with their recommended adhesives seem to be suitable for stabilizing endodontically treated teeth (ETT). Although there was no significant difference, the highest fracture load was found for ETT with access cavities restored with Venus Diamond, followed by direct restoration with Estelite Sigma Quick and Tetric Evoceram. Only restorations with Estelite Sigma Quick showed grade 1 fractures. For future studies, investigations with distinctly larger defect sizes are planned.

## Figures and Tables

**Table 1:** Material specifications of resin composites used for direct restoration of endodontic access cavities.

Resin Composite	Base Monomers	Filler Content	Filler Type
Tetric Evo Ceram^®^	Bis-GMA, Bis-EMA, UDMA	60–61 vol% 82.5 wt%	barium glass particles Ø 0.6 μm
Venus Diamond^®^	TCD-DI-HEA, UDMA	64 vol% 80–82 wt%	barium aluminium fluoride glass particles Ø 5 nm–20 μm
Grandio^®^	Bis-GMA, Bis-EMA, TEGDMA	71.4 vol% 87 wt%	glass ceramic particles Ø 1 μm, silicon dioxide nanoparticles Ø 20–40 nm
Estelite^®^ Sigma Quick (lot…)	Bis-GMA, TEGDMA	71 vol% 82 wt%	pre-polymer Ø 2 μm, silicon-zirconia- particles Ø 0.2 μm

**Table 2:** Manufacturer recommended resin composite and adhesive combinations. Etch and rinse with 35% ortho-phosphoric acid (light blue) was applied for Syntac classic^®^, iBond total etch^®^ and Solobond M^®^. Application of the adhesive Bond Force^®^ required no additional cavity preconditioning (self-etch mode).

Resin Composite	Adhesive System	Mode of Etching	Primer	Adhesive	Bonding
Tetric Evo Ceram^®^	Syntac classic^®^	ortho-phosphoric acid (Etch and Rinse)	Syntac Primer^®^	Syntac Adhäsiv^®^	Heliobond^®^
Venus Diamond^®^	iBond total etch^®^	ortho-phosphoric acid (Etch and Rinse)	iBond total etch^®^		
Grandio^®^	Solobond M^®^	ortho-phosphoric acid (Etch abd Rinse)	Solobond M^®^		
Estelite^®^ Sigma Quick	Bond Force^®^	Bond Force^®^			

## Data Availability

The original contributions presented in the study are included in the article, further inquiries can be directed to the corresponding author.
